# miR-146a, an IL-1β responsive miRNA, induces vascular endothelial growth factor and chondrocyte apoptosis by targeting Smad4

**DOI:** 10.1186/ar3798

**Published:** 2012-04-16

**Authors:** Jing Li, Jingang Huang, Liming Dai, Degang Yu, Qian Chen, Xiaoling Zhang, Kerong Dai

**Affiliations:** 1 The Key Laboratory of Stem Cell Biology, Institute of Health Sciences, Shanghai Institutes for Biological Sciences, Chinese Academy of Sciences & Shanghai Jiao Tong University School of Medicine, China, 200025; 2 Shanghai Key Laboratory of Orthopaedic Implant, Department of Orthopaedic Surgery, Shanghai Ninth People's Hospital, Shanghai Jiao Tong University School of Medicine, China, 200011; 3 Cell and Molecular Biology Laboratory, Department of Orthopaedics, Warren Alpert Medical School of Brown University/Rhode Island Hospital, Providence, USA, RI 02903

## Abstract

**Introduction:**

miR-146a is one of the first identified miRNAs expressed differentially in osteoarthritis (OA) cartilage. However, the role it plays in OA pathogenesis is not clear. The aim of this study is to identify a molecular target of miR-146a, thereby elucidating its function in chondrocytes during OA pathogenesis.

**Methods:**

Primary chondrocytes from Sprague-Dawley rats were treated with IL-1β before the expression levels of miR-146a, Smad4 and vascular endothelial growth factor (VEGF) were quantified by real-time PCR and/or western blotting. The effect of miR-146a on cellular response to transforming growth factor (TGF)-β1 was quantified by a luciferase reporter harboring TGF-β1 responsive elements and by extracellular signal-regulated kinase assay. The effect of miR-146a on apoptosis was quantified by the TUNEL assay. OA pathogenesis was surgically induced with joint instability in rats, evaluated by histopathological analysis with safranin O staining, and the expression levels of miR-146a, Smad4, and VEGF were quantified using real-time PCR and/or immunohistochemistry.

**Results:**

IL-1β treatment of chondrocytes increased the expression levels of miR-146a and VEGF and decreased the levels of Smad4 in a time-dependent manner. miR-146a upregulated VEGF expression and downregulated Smad4 expression in chondrocytes, while a miR-146a inhibitor acted in a converse manner. Smad4, a common mediator of the TGF-β pathway, is identified as a direct target of miR-146a by harboring a miR-146a binding sequence in the 3'-UTR region of its mRNA. Mutation of the binding sequence significantly relieved the inhibition of the Smad4 reporter activity by miR-146a. Furthermore, miR-146a upregulation of VEGF is mediated by Smad4. Expression of miR-146a led to a reduction of cellular responsiveness to TGF-β and an increase of apoptosis rate in chondrocytes. *In vivo*, cartilage from surgically induced OA rats displayed higher levels of miR-146a and VEGF compared with the sham group. In contrast, Smad4 expression level was lower in the OA group than the sham group.

**Conclusion:**

IL-1β responsive miR-146a is overexpressed in an experimentally induced OA model, accompanied by upregulation of VEGF and downregulation of Smad4 *in vivo*. miR-146a may contribute to OA pathogenesis by increasing VEGF levels and by impairing the TGF-β signaling pathway through targeted inhibition of Smad4 in cartilage.

## Introduction

miRNAs have emerged as a novel class of gene regulators in both animals and plants that regulate the expression of more than one-third of human genes post-transcriptionally [1]. There is accumulating evidence that miRNAs are multifunctional mediators in regulating physiological processes, including development, proliferation, differentiation, and apoptosis [2,3]. Although most of them are widely distributed, the expression of some miRNAs exhibits cell-type-specific, tissue-specific, and developmental-stage-specific patterns [2]. miRNAs have also been reported to influence pathological processes, such as cancer, diabetes, and cardiovascular diseases [3]. miRNAs act as key regulators in various types of diseases because dysregulation of specific miRNAs occurs prevalently under disease conditions [4,5]. Several miRNAs have been identified, showing differential expression patterns between osteoarthritis (OA) and normal cartilage, and their postulated functions are related to inflammatory and catabolic changes in OA [6]. miR-146a is one of the first identified miRNAs associated with OA cartilage [7]. miR-146a is expressed in all layers of human articular cartilage, especially in the superficial zone, and its expression is upregulated in OA [7]. However, the exact etiological mechanism of miR-146a in OA pathogenesis is not clear.

The imbalance of cartilage homeostasis between catabolic and anabolic activities contributes to the etiology of OA [8]. A number of cytokines take part in this process. Proinflammatory cytokines such as IL-1β and TNFα are catabolic factors that lead to the breakdown of articular cartilage [9], while anabolic factors such as transforming growth factor (TGF)-β superfamily members have been shown to exert a protective effect in OA [10]. Smad4, a common mediator of the TGF-β pathway (co-Smad), plays an important role in transducing TGF-β signals by forming intracellular signaling complexes with phosphorylated receptor-regulated Smads (R-Smads). The complexes then translocate into the nucleus where they participate in the initiation or repression of gene expression, thereby regulating the transcription of target genes [11]. In contrast, IL-1β functions as a main catabolic factor in the OA process and the elevation of IL-1β causes degradation of the cartilage extracellular matrix [12].

In this study we present evidence that miR-146a is upregulated in articular chondrocytes in response to IL-1β treatment *in vitro *and by destabilization of the knee joints *in vivo*, and that Smad4 is a direct target of miR-146a. We find that the miR-146a inhibition of Smad4 results in upregulation of vascular endothelial growth factor (VEGF) and apoptosis of chondrocytes. Conversely, inhibiting miR-146a or overexpressing Smad4 reduces VEGF expression in chondrocytes. Furthermore, we demonstrate that miR-146a upregulation *in vivo *is accompanied by downregulation of Smad4 and upregulation of VEGF in a surgically induced OA model of Sprague-Dawley rats. Together, these findings suggest that dysregulation of miR-146a may contribute to OA pathogenesis by inhibiting Smad4, a key component in the anabolic TGF-β pathway, by stimulating VEGF in the angiogenesis, chondrocyte hypertrophy, and extracellular matrix degradation pathways, and by inducing chondrocyte death.

## Materials and methods

### Primary cell culture

Primary chondrocytes were isolated from the femoral condyles and tibial plateau of male Sprague-Dawley rats (160 to 180 g). Rat articular cartilage was cut into small fragments, followed by digestion first with 0.25% trypsin (Gibco Invitrogen, Carlsbad, CA, USA) for 30 minutes at 37°C and then with 0.2% collagenase (Sigma-Aldrich, St Louis, MO, USA) for 5 hours at 37°C. After dissociation, the cell suspension was filtered through a 40 μm cell strainer (BD Falcon, Bedford, MA, USA), and cells were collected by centrifugation at 800 × *g *for 10 minutes. Chondrocytes were then resuspended in DMEM/F-12 medium (Gibco Invitrogen) supplemented with 10% fetal bovine serum (Gibco Invitrogen). Primary chondrocytes were cultured according to a previous method [13]. Briefly, chondrocytes were placed in monolayer culture in six-well plates (for RNA) or 12-well plates (for protein) in DMEM/F-12 medium containing 10% fetal bovine serum. Transfection experiments were performed 1 day after seeding. Primary chondrocytes used in the experiments were either freshly isolated or were at passage 1. Either freshly isolated or at passage 1, these chondrocytes do not express Col I - a marker of dedifferentiation - as determined by real-time RT-PCR. The observed effects of miR-146a are identical in chondrocytes at the freshly isolated and passage 1 stage.

### miRNA microarray

The miRNA expression profiles of the rat chondrocytes treated with IL-1β (10 ng/ml; R&D Systems, Minneapolis, MN, USA) at various time points were determined by miRNA microarray analysis using the μParaflo™ microfluidic chips (LC Sciences, Houston, TX, USA), which were based on Sanger miRBase Release 17.0 [14]. Total RNA was size-fractionated and the small RNAs (< 300 nucleotides) isolated were 3'-extended with a poly(A) tail. Hybridization was performed overnight. Data were analyzed by first subtracting the background and then normalizing the signals using a LOWESS filter (locally-weighted regression) [15]. Normalized data were further analyzed by one-way analysis of variance followed by a Student-Newman-Keuls multiple comparison test. miRNAs with *P *< 0.01 were considered differentially expressed.

### Construction of plasmids and site-directed mutagenesis

For plasmid DNA and miRNA co-transfection, primary chondrocytes were transfected using the Human Chondrocyte Nucleofector kit (Amaxa, Cologne, Germany) following the manufacturer's instructions [16]. The miR-146a expression plasmid was created as previously described [17]. Briefly, the precursor sequence for miR-146a was amplified through PCR using genomic DNA as the template, and the PCR products were cloned into the pSuper vector (Oligoengine, Seattle, WA, USA). Fragments harboring the 3' UTR of Smad4 were cloned into the *Xba*I site of the pGL3-control vector (Promega, Madison, WI, USA) using the following primers: sense, 5'-CCGCTCGAGTGAAGGAATCATTCCAGTGCTAG-3'; and antisense, 5'-TGCTCTAGACTTGGTAAAATTAACTCACCCACA-3'. The mutated 3' UTR luciferase reporter plasmid was generated by site-directed mutagenesis using the QuikChange site-directed mutagenesis kit (Stratagene, La Jolla, CA, USA). The following primers were used: sense, 5'-TTAAAGGCAGAGAACAAGAGAAAGTTAATTCACC-3'; and antisense, 5'-GGTGAATTAACTTTCTCTTGTTCTCTGCCTTTAA-3'. All sequences of the amplified products were confirmed by DNA sequencing.

### Luciferase reporter assay

All plasmids for transfection were prepared using the QIAGEN plasmid purification kit (QIAGEN, Hilden, Germany). HEK293T cells were transiently transfected using Lipofectamine 2000 (Invitrogen, Carlsbad, CA, USA) according to the manufacturer's instructions, and pRL-SV40 vector (Promega) was used as a control for transfection efficiency. Twenty-four hours after transfection, cells were lysed, and Firefly and Renilla luciferase activities were measured using the Dual-Luciferase Reporter Assay System (Promega) according to the manufacturer's protocol. C5.18 cells were co-transfected with miR-146a mimics (GenePharma, Shanghai, China) and p3TP-lux using DharmaFECT Duo transfection reagent (Dharmacon Inc., Lafayette, CO, USA). The p3TP-lux plasmid was a kind gift from Dr Regis J. O'Keefe (Center for Musculoskeletal Research, University of Rochester, Rochester, New York, USA). Twelve hours after transfection, the cells were serum starved for 12 hours followed by 4 hours treatment with or without TGF-β1 (10 ng/ml, R&D Systems). Cell lysates were extracted and luciferase activities were measured using the Dual-Luciferase Reporter Assay System (Promega). Each experiment was repeated at least three times.

### RNA and quantitative real-time PCR

Total RNA, including miRNA, was extracted using the miRNeasy Mini Kit (QIAGEN) according to the manufacturer's instructions. Then 1 μg total RNA was reverse-transcribed with a specific stem-loop primer for miRNA and with a random primer for mRNA, respectively. After RT reaction, real-time PCR was performed by an ABI 7900HT system using SYBR Premix Ex Taq™ (Takara, Madison,. WI, USA). β-actin and small nuclear RNA U6 were used as internal controls for cDNA and miRNA, respectively. Primer sequences used for real-time PCR are presented in Table 1.

**Table 1:** Primer sequences used for real-time PCR

	Sense primer	Antisense primer
Rat β-actin [GenBank:NM_031144.2]	5'-CTCTTCCAGCCTTCCTTCCT-3'	5'-TCATCGTACTCCTGCTTGCT-3'
Rat U6 [GenBank:K00784]	5'-CTCGCTTCGGCAGCACA-3'	5'-AACGCTTCACGAATTTGCGT-3'
Rat Smad4 [GenBank:NM_019275.2]	5'-CCACCAACTTCCCCAACATT-3'	5'-TGCAGTCCTACTTCCAGTCCAG-3'
Rat VEGF [GenBank:NM_031836.2]	5'-TTGAGACCCTGGTGGACATCT-3'	5'-CTCCTATGTGCTGGCTTTGG-3'

VEGF, vascular endothelial growth factor.

### Western blotting

Whole-cell lysates were prepared with ice-cold lysis buffer (50 mM Tris-HCl, pH 7.4, 150 mM NaCl, 1% NP-40, and 0.1% sodium dodecyl sulfate) supplemented with protease inhibitors (Complete Tablet; Roche, Mannheim, Germany). Proteins were size-fractionated by SDS-PAGE and transferred to a PVDF membrane (Hybond-P; Amersham Biosciences, Amersham, UK). Membranes were hybridized with antibodies against Smad4 (1:1,000; Santa Cruz, Santa Cruz, CA, USA), VEGF (1:1,000; Santa Cruz), extracellular signal-regulated kinase (ERK) 1/2 (1:1,000; Cell Signaling Technology, Danvers, MA, USA), phospho-ERK1/2 (1:1,000; Cell Signaling Technology) and GAPDH (1:5,000; Kangcheng, Shanghai, China). Densitometric analysis of immunoblots was performed using the ImageJ software provided by the National Institutes of Health (Developed by National Institutes of Health, Bethesda, Maryland, USA).

### Smad4 knockdown by siRNA

RNA interference was performed using siGENOME SMARTpool siRNA (Dharmacon Inc.) targeting rat Smad4. Transfection for primary chondrocytes was carried out using Lipofectamine RNAiMAX reagent (Invitrogen) according to the manufacturer's protocol.

### TUNEL assay

Chondrocytes were fixed for 20 minutes at room temperature with 4% paraformaldehyde in PBS 48 hours post transfection, and apoptosis was assessed using the In Situ Cell Death Detection Kit - Fluorescein (Roche) according to the manufacturer's instructions. The number of TUNEL-positive cells (green) and the number of Hoechst 33342-positive cells (blue nuclear stain) were visually counted. All samples were analyzed with at least three independent replicates, and five fields from each replicate were randomly selected for counting the TUNEL-positive cells and the Hoechst 33342-positive cells. The observer who performed the cell counts and immunofluorescence quantitation was blinded to the types of the samples.

### Surgical induction of osteoarthritis

Animal handling and experimental procedures were performed following approval from the Institute of Health Sciences Institutional Animal Care and Use Committee. Eight-week-old male Sprague-Dawley rats (200 g) were randomized into two groups of 20 rats each. OA was induced by medial collateral ligament transection and medial meniscal tear of the knee joints, as previously described [18]. Briefly, animals were anesthetized and surgery was performed to transect the medial collateral ligament and to cut the medial meniscus through the full thickness to induce joint destabilization of the right knee. Sham animals underwent the same surgical procedure without any ligament transection or meniscal tear. After surgery, each rat was given penicillin once per day for the first 3 days. Animals were sacrificed at 8 weeks post surgery, and samples of the knee joints were collected for further molecular and histological analyses.

### Histology and immunohistochemistry

Knee joints from the model animals were fixed overnight with 4% paraformaldhyde in PBS and then embedded in paraffin. Tissue sections (5 μm) were deparaffinized in xylene, serially rehydrated in ethanol, and washed with PBS. Sections were stained with safranin O/fast green to identify proteoglycan loss [19]. For immunohistochemistry, sections in 10 mM sodium citrate buffer (pH 6.0) were heated in a microwave oven and kept at 95°C for 10 minutes. Slides were cooled for 30 minutes at room temperature after antigen unmasking. Endogenous peroxidase activity was blocked with 3% hydrogen peroxide, followed by rinsing several times in PBS. After blocking nonspecific protein binding with 5% BSA in PBS for 30 minutes at room temperature, sections were incubated overnight at 4°C with primary antibodies against Smad4 (Santa Cruz) and VEGF (Santa Cruz). The slides were rinsed in PBS and then incubated with secondary antibody (EnVision detection kit, Peroxidase/DAB, Rabbit/mouse; Dako Cytomation, Carpinteria, CA, USA) according to the manufacturer's protocol. Sections were counterstained with Mayer's hematoxylin (Sigma, St. Louis, MO, USA). After washing, the slides were stained with 3,3'-diaminobenzidine tetrahydrochloride (EnVision detection kit, Peroxidase/DAB, Rabbit/mouse; Dako Cytomation). Staining with normal IgG and staining without primary antibodies were also performed as negative controls. For immunohistochemistry, sections were quantified using ImagePro Plus version 5.0 (Media Cybernetics, Bethesda, MD, USA). Three fields of view per section were analyzed from each animal. Mean values and variances of Smad4-positive and VEGF-positive cells in each group were calculated from 20 animals per group.

### Statistical analysis

Results are expressed as mean ± standard deviation. Statistical analysis was carried out using Student's *t *test between two groups or one-way analysis of variance followed by Student-Newman-Kuels test for multiple comparisons. *P *< 0.05 were considered statistically significant.

## Results

### IL-1β treatment increases expression of miR-146a and VEGF and decreases Smad4 expression in chondrocytes

To identify the miRNAs involved in pathogenesis of OA, we screened for miRNAs responsive to treatment of the proinflammatory cytokine IL-1β (10 ng/ml) in primary rat chondrocytes. This is an established cell culture model to mimic inflammation and other molecular events related to the progression of OA in chondrocytes [20]. Expression of miRNAs in IL-1β-stimulated chondrocytes was investigated by microarray analysis [GEO:GSE33310] [21]. A series of miRNAs changed their expression levels in response to IL-1β treatment (Figure S1 in Additional file 1). Of particular interest, miR-146a was chosen for further investigation because previous studies have revealed that miR-146a mediates inflammation response [22], and its expression is higher in OA cartilage than in normal cartilage [7].

**Figure 1:**
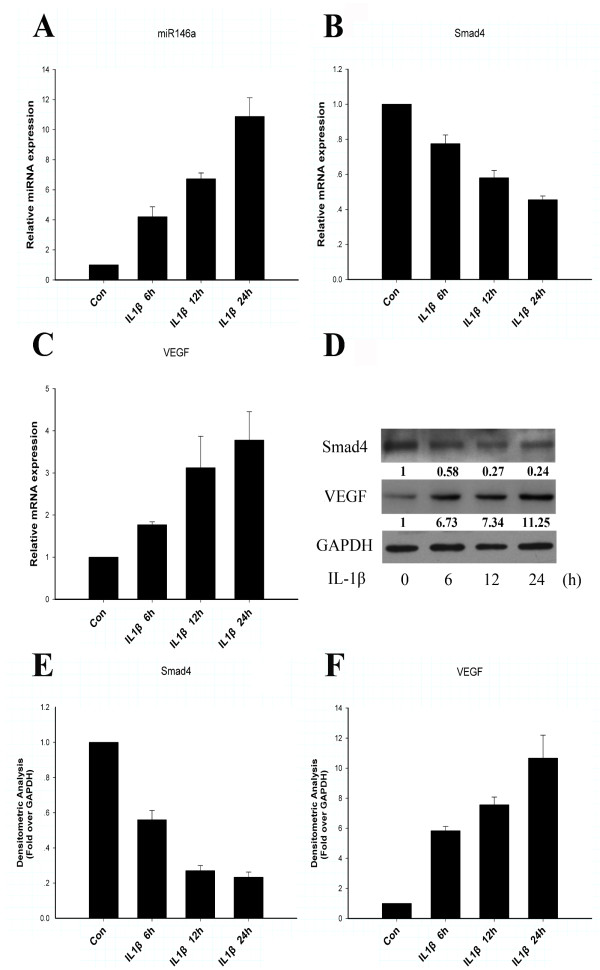
**IL-1β stimulates miR-146a and vascular endothelial growth factor along with downregulation of Smad4**. Primary rat chondrocytes were treated with IL-1β (10 ng/ml) for the indicated time periods. Expression levels of miR-146a, Smad4, and vascular endothelial growth factor (VEGF) were monitored by quantitative real-time PCR and western blotting. **(A) **miR-146a expression was significantly increased over a 24-hour time course. **(B) **mRNA levels of Smad4 were reduced after treatment with IL-1β. **(C) **VEGF was upregulated by IL-1β. Values are the mean ± standard deviation (SD) of three independent experiments. **(D) **Protein levels of Smad4 and VEGF were respectively decreased and increased in chondrocytes stimulated with IL-1β. The relative expression levels of protein are shown at the bottom of the bands as normalized by the GAPDH level. **(E) **Densitometric analysis of immunoblot band intensities for Smad4 normalized by GAPDH. Data are mean ± SD of three independent experiments. **(F) **Densitometric analysis of immunoblot band intensities for VEGF normalized by GAPDH. Data are mean ± SD of three independent experiments. Control samples (Con) were not treated with IL-1β.

Treatment of IL-1β rapidly induced miR-146a within 6 hours in primary rat chondrocytes, and its expression gradually increased over a 24-hour time course (Figure 1A), which is consistent with the microarray results. In parallel with the increase of miR-146a level, IL-1β treatment stimulated VEGF mRNA (Figure 1C) and protein levels (Figure 1D) in a time-dependent manner. In contrast, IL-1β treatment inhibited Smad4 mRNA (Figure 1B) and protein levels (Figure 1D) in a time-dependent manner.

### miR-146a directly inhibits Smad4 expression through a seed site in the 3'-UTR of Smad4 mRNA

**Figure 2:**
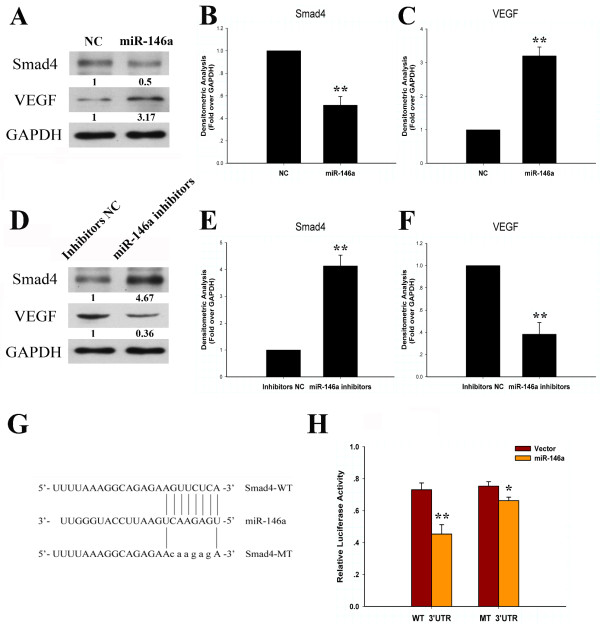
**Smad4 is a direct target of miR-146a**. **(A) **Overexpression of miR-146a inhibits Smad4 expression and increases vascular endothelial growth factor (VEGF) expression in primary chondrocytes, as assayed by immunoblotting. NC, negative control. **(B) **Densitometric analysis of immunoblot band intensities for Smad4 normalized by GAPDH. Data are mean ± standard deviation (SD) of three independent experiments. **(C) **Densitometric analysis of immunoblot band intensities for VEGF normalized by GAPDH. Data are mean ± SD of three independent experiments. **(D) **On the protein level, knockdown of miR-146a results in the upregulation of Smad4 and the downregulation of VEGF. Relative expression levels of protein shown at the bottom of the bands as normalized by GAPDH level. **(E) **Densitometric analysis of immunoblot band intensities for Smad4 normalized by GAPDH. Data are mean ± SD of three independent experiments. **(F) **Densitometric analysis of immunoblot band intensities for VEGF normalized by GAPDH. Data are mean ± SD of three independent experiments. **(G) **Schematic representation of the Smad4 3' UTR indicating the binding site of miR-146a. WT, wildtype; MT, mutant. **(H) **miR-146a inhibits Smad4 3' UTR luciferase activity. HEK293T cells were transfected with either pGL3 luciferase vector containing a fragment of Smad4 3' UTR harbouring binding sites for miR-146a, or the corresponding mutant constructs. Ectopic expression of miR-146a led to a remarkable reduction of the luciferase activity of reporter with the wildtype 3' UTR but not that of the mutant reporter. Values are mean ± SD of three independent experiments. *P < 0.05 versus vector. **P < 0.01 versus vector.

To determine whether miR-146a regulates the expression of Smad4 and VEGF, we transfected miR-146a into primary chondrocytes. Overexpression of miR-146a inhibited Smad4 protein levels and stimulated VEGF protein levels (Figure 2A). Conversely, transfection of a miR-146a inhibitor stimulated Smad4 protein levels and inhibited VEGF protein levels in chondrocytes (Figure 2D). miR-146a thus regulates the expression of Smad4 and VEGF in an opposite manner.

Using miRNA target prediction software [1], we identified a potential miR-146a binding sequence in the 3' UTR of Smad4 (Figure 2G). To determine whether miR-146a inhibits Smad4 expression through this seed sequence, we constructed luciferase reporter plasmids harboring the wildtype 3' UTR and the mutant 3' UTR in which the putative miR-146a binding site is mutated (Figure 2G). While the reporter activity of the wildtype 3' UTR is significantly inhibited by miR-146a, this inhibition is greatly reduced in the mutant 3' UTR (Figure 2H). Smad4 is thus a direct target of miR-146a.

### IL-1β regulates Smad4 and VEGF expression through miR-146a

**Figure 3:**
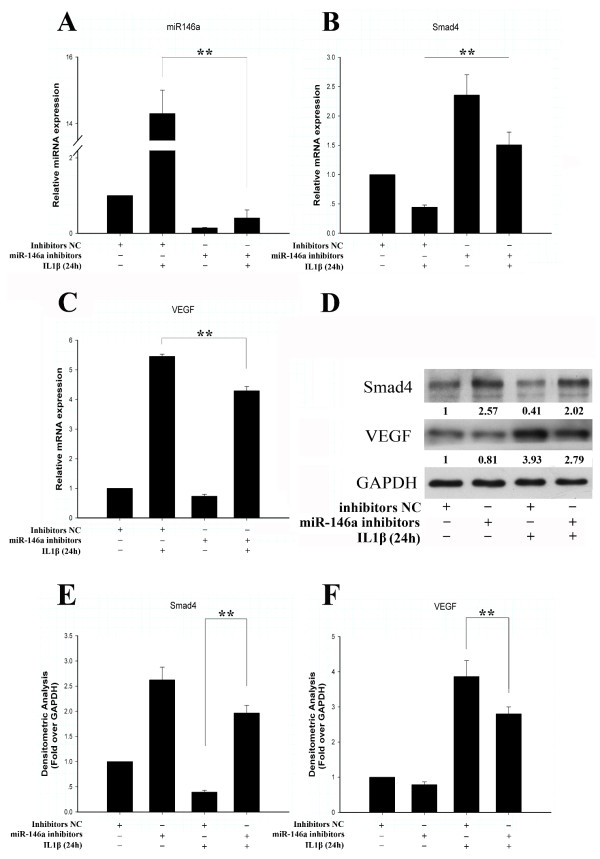
**miR-146a suppression impairs IL-1β downregulation of Smad4 and upregulation of vascular endothelial growth factor**. Chondrocytes were transfected with miR-146a inhibitors and treated with or without IL-1β for 24 hours. **(A) **miR-146a inhibitors decreased IL-1β-induced upregulation of miR-146a. NC, negative control. **(B) **The inhibitory effect of IL-1β on Smad4 expression was counteracted by miR-146a inhibitors. **(C) **Vascular endothelial growth factor (VEGF) expression was significantly reduced by miR-146a inhibitors with IL-1β treatment at the RNA level. Values are the mean ± standard deviation (SD) of at least three independent experiments. **P < 0.01. **(D) **On the protein level, miR-146a inhibitors counteracted IL-1β-induced downregulation of Smad4 and upregulation of VEGF. The relative expression levels of protein are shown at the bottom of the bands as normalized by GAPDH level. **(E) **Densitometric analysis of immunoblot band intensities for Smad4 normalized by GAPDH. Data are mean ± SD of three independent experiments. **(F) **Densitometric analysis of immunoblot band intensities for VEGF normalized by GAPDH. Data are mean ± SD of three independent experiments.

To elucidate the role of miR-146a in mediating IL-1β signaling, we used a specific miR-146a hairpin inhibitor to block its expression. Chondrocytes were treated with IL-1β for 24 hours in the presence or absence of the miR-146a inhibitor. Knockdown of endogenous miR-146a with the inhibitor significantly suppressed the IL-1β upregulation of miR-146a expression (Figure 3A). While IL-1β treatment inhibited Smad4 mRNA levels, transfection of the miR-146a inhibitor markedly increased Smad4 mRNA despite the presence of IL-1β (Figure 3B). While IL-1β treatment greatly increased the VEGF mRNA levels, the miR-146a inhibitor significantly reduced this increase (Figure 3C). Knockdown of miR-146a caused similar effects on the IL-1β regulation of Smad4 and VEGF protein levels as on their mRNA levels (Figure 3D). miR-146a is thus involved in IL-1β regulation of Smad4 and VEGF expression.

### Upregulation of VEGF by miR-146a is mediated by Smad4

**Figure 4:**
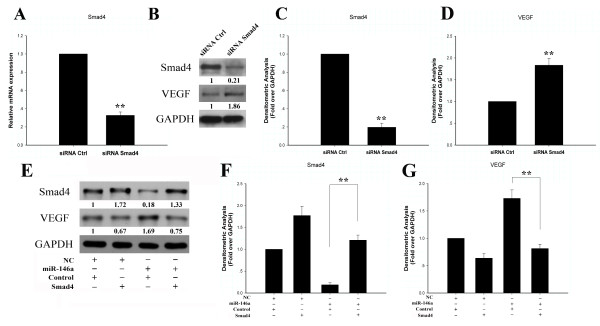
**Vascular endothelial growth factor production is regulated by Smad4**. Knockdown of Smad4 by RNAi increased vascular endothelial growth factor (VEGF) expression at the protein level. Chondrocytes were transfected with siRNA specific for Smad4 or with control (Ctrl) siRNA. **(A) **siRNA targeting Smad4 efficiently reduced endogenous Smad4 expression at the RNA level. Values are the mean ± standard deviation (SD) of at least three independent experiments. **P < 0.01. **(B) **Inhibition of Smad4 increased the protein level of VEGF. **(C) **Densitometric analysis of immunoblot band intensities for Smad4 normalized by GAPDH. Data are mean ± SD of three independent experiments. **(D) **Densitometric analysis of immunoblot band intensities for VEGF normalized by GAPDH. Data are mean ± SD of three independent experiments. **(E) **Co-transfection of miR-146a and Smad4 expression plasmid reversed miR-146a-mediated upregulation of VEGF. Relative expression levels of protein shown at the bottom of the bands as normalized by GAPDH level. NC, negative control. **(F) **Densitometric analysis of immunoblot band intensities for Smad4 normalized by GAPDH. Data are mean ± SD of three independent experiments. **(G) **Densitometric analysis of immunoblot band intensities for VEGF normalized by GAPDH. Data are mean ± SD of three independent experiments.

To determine whether Smad4 mediates the upregulation of VEGF by miR-146a, RNA interference with Smad4 siRNA was performed in rat chondrocytes. Chondrocytes were transfected with siRNA against Smad4. This Smad4 siRNA transfection reduced the levels of both Smad4 mRNA (Figure 4A) and protein (Figure 4B). Knockdown of Smad4 increased VEGF protein levels (Figure 4B), while overexpression of Smad4 significantly reduced miR-146a stimulation of VEGF protein levels (Figure 4E). Smad4 thus mediates upregulation of VEGF by miR-146a.

### miR-146a attenuates TGF-β signaling pathway

**Figure 5:**
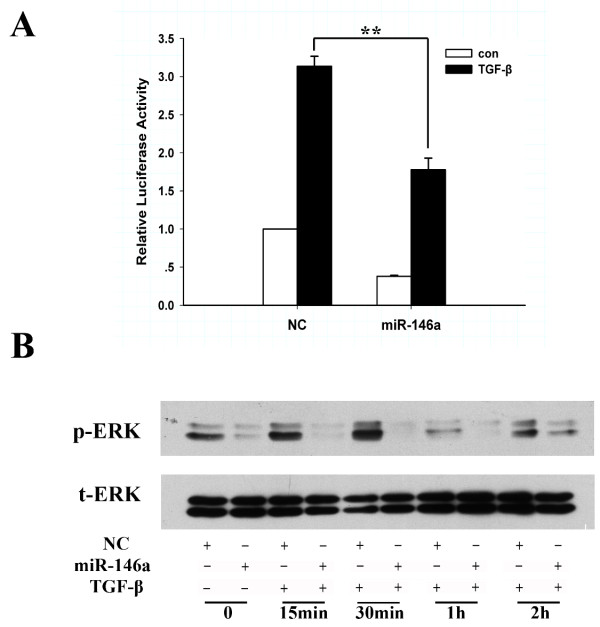
**miR-146a attenuates the transforming growth factor-β signaling pathway**. **(A) **C5.18 cells were co-transfected with miR-146a and p3TP-lux reporter plasmid followed by treatment with or without exogenous transforming growth factor (TGF)-β1 (10 ng/ml) for 4 hours. The cell lysates were obtained to determine the luciferase activity. Values are the mean ± standard deviation of at least three independent experiments. **P < 0.01. NC, negative control. **(B) **Overexpression of miR-146a blocked TGF-β1-stimulated activation of extracellular signal-regulated kinase (ERK). Chondrocytes were transfected with miR-146a mimics, and, after serum starvation, cells were treated with TGF-β1 (10 ng/ml) for the indicated periods. Cell lysates were analyzed for phosphorylated and total ERK levels in chondrocytes.

Because Smad4 is a common mediator of the TGF-β signaling pathway, we next addressed the question of whether miR-146a affects the cellular responses to TGF-β. C5.18 cells were co-transfected with miR-146a and p3TP-luciferase reporter plasmid (p3TP-lux, possessing TGF-β response elements) followed by treatment with TGF-β1 (10 ng/ml). As shown in Figure 5A, overexpression of miR-146a led to a decrease in both basal and TGF-β1-stimulated activity of the p3TP-luciferase reporter, suggesting that miR-146a significantly inhibits TGF-β signaling transduction. To further investigate the role of miR-146a in TGF-β signaling, we conducted a time-course study of ERK activation by TGF-β1 in chondrocytes transfected with miR-146a. Western blot analysis revealed time-dependent activation of ERK with maximal activation occurring at 30 minutes post treatment (Figure 5B). Overexpression of miR-146a reduced the levels of phospho-ERK 1/2 at all time points (Figure 5B), whereas the total ERK levels remained relatively constant (Figure 5B).

### miR-146a increases apoptosis in chondrocytes

**Figure 6:**
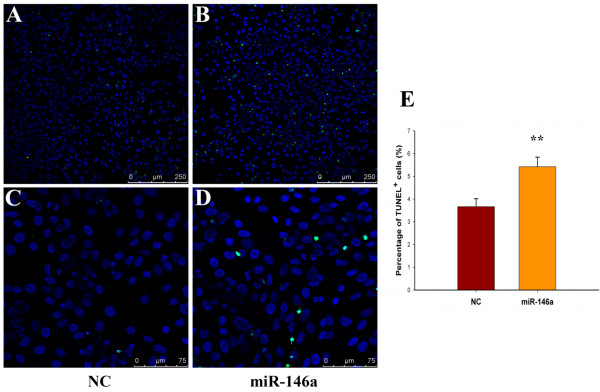
**miR-146a leads to apoptosis in chondrocytes**. **(A) to (D) **TUNEL assays were performed 2 days following the addition of negative control (NC) or miR-146a to chondrocytes. miR-146a increases apoptosis in chondrocytes. The nuclei of apoptotic cells are labeled by TUNEL with green fluorescence. All cell nuclei are counterstained by Hoechst 33342 (blue). Original magnification: (A), (B) ×200; (C), (D) ×600. **(E) **Quantification of the percentage of TUNEL-positive cells shows a 40.3% increase in the percentage of apoptotic cells in chondrocytes transfected with miR-146a. **P < 0.01.

Since IL-1β stimulates apoptosis in chondrocytes [23] and the loss of cellularity is a hallmark of OA cartilage [24], we examined whether the expression of miR-146a affects chondrocyte apoptosis. Overexpression of miR-146a in chondrocytes caused a significant increase of the percentage of TUNEL-positive cells (Figure 6), indicating that miR-146a takes part in mediating IL-1β-induced apoptosis in chondrocytes.

### Co-regulation of miR-146a with Smad4 and VEGF in OA cartilage *in vivo*

**Figure 7:**
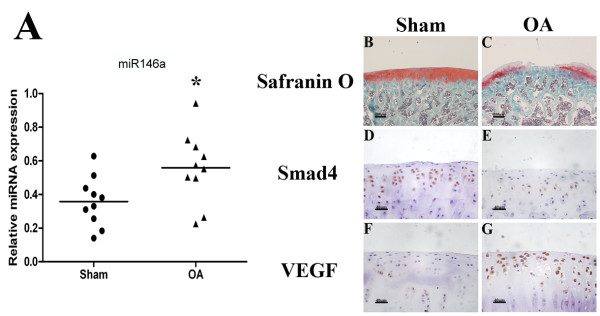
**miR-146a and Smad4 are expressed reciprocally in surgically induced osteoarthritis in rats**. **(A) **The level of miR-146a increases in cartilage from the osteoarthritis (OA) group compared with the sham group. Values are the mean ± standard deviation of 10 animals per group. *P < 0.05. Femoral condyles from OA and Sham rats were stained with safranin O and subjected to immunohistochemistry for Smad4 and vascular endothelial growth factor (VEGF). **(B), (C) **Femoral condyles from OA rats exhibited reduced safranin O staining. **(D), (E) **Cartilage from normal and osteoarthritic rats were immunostained for Smad4 protein. **(F), (G) **Cartilage from normal and osteoarthritic rats were immunostained for VEGF protein. Original magnification: (B), (C) ×40; (D) to (G) ×400.

To determine whether expression of miR-146a, Smad4 and VEGF is co-regulated in OA cartilage *in vivo*, we surgically induced OA through joint instability in Sprague-Dawley rats (Figure 7B, C). The expression of miR-146a was significantly upregulated in OA cartilage compared with normal cartilage (Figure 7A). Immunohistochemical analysis showed a decrease of Smad4-positive cells (Figure 7D, E) and an increase of VEGF-positive cells (Figure 7F, G) in OA cartilage than in normal cartilage (sham). The percentage of chondrocytes positive for Smad4 was substantially decreased in the OA group (31.5 ± 5.1%) compared with the sham group (61.1 ± 4.6%), while the percentage of VEGF-positive cells in the sham and OA groups (8.3 ± 2.1% and 63.7 ± 5.4%, respectively) indicated a statistically significant increase in OA cartilage (*P *< 0.01). The induction of miR-146a expression in OA cartilage is thus correlated with the upregulation of VEGF and the downregulation of Smad4 in rat joints with surgically induced OA.

## Discussion

miR-146a is one of the first identified miRNAs upregulated in human OA cartilage. However, it was not clear whether this is a coincidence or miR-146a plays a role in OA pathogenesis. We provide several lines of evidence here to demonstrate that miR-146a may be an important regulator in OA.

First, we demonstrate for the first time that miR-146a is upregulated by experimentally induced OA pathogenesis in a well-established OA animal model of Sprague-Dawley rats *in vivo*. The induction of miR-146a expression in articular cartilage is thus caused by OA. In addition to miR-146a, other miRNAs may also play important roles in OA pathogenesis: miR-140, a cartilage-specific miRNA, regulates gene expression of ADAMTS-5 in chondrocytes [25]; and *miR-140*^-/- ^mice display an OA-like phenotype [26]. miR-140 may also be involved in the formation and maintenance of cartilage through targeting HDAC4 [27]. In addition, miR-27a affects the expression of matrix metalloproteinase (MMP)-13 and IGFBP-5 [28], and miR-27b inhibits the IL-1β-induced upregulation of MMP-13 in human osteoarthritic chondrocytes [29].

Second, we demonstrate that miR-146a is induced by IL-1β treatment of chondrocytes in a time-dependent manner *in vitro*. We focused our study on miR-146a after it came up in our screening for IL-1β upregulated miRNAs in chondrocytes. Our observation and the previous literature suggest that the responsiveness to IL-1β and/or other inflammatory cytokines is a hallmark of miR-146a. The expression of miR-146a/b was elevated after treatment with lipopolysaccharide and proinflammatory mediators [22]. Stanczyk and colleagues reported that the expression of miR-146 is increased in rheumatoid arthritis synovial fibroblasts [30]. Nakasa and colleagues reported increased miR-146a/b expression in synovial tissue from rheumatoid arthritis patients [31]. miR-146a operates as a negative regulator in innate immunity by affecting IL-1R-associated kinase-1 and TNF-receptor-associated factor 6. In human OA tissue samples, miR-146a may be involved in both proinflammatory cytokine response and modulation [7,32].

Third, we demonstrate that miR-146a is induced by joint instability resulting from medial collateral ligament transection and medial meniscal tear of the knee joints *in vivo*. The inductive factors for miR-146a may be more complex *in vivo*. In addition to the proinflammatory cytokines resulting from the medial collateral ligament transection and medial meniscal tear, mechanical instability is also a major cause of OA pathogenesis in this animal model [18]. Mechano-responsive miRNAs are beginning to be identified in chondrocytes. miR-365 is the first identified mechanically responsive miRNA in chondrocytes, which regulates chondrocyte differentiation through inhibiting HDAC4 [33]. In addition, miR-222 was postulated as a potential regulator of the articular cartilage mechanotransduction pathway, since its expression patterns in articular cartilage are higher in the weight-bearing anterior medial condyle as compared with the posterior nonweight-bearing medial condyle [34]. It remains to be tested whether miR-146a is responsive to alteration of mechanical load in addition to proinflammatory cytokine.

Fourth, we have for the first time identified a direct molecular target of miR-146a in chondrocytes. We show that the expression levels of Smad4, a key transcription factor mediating the TGF-β family member signaling pathway, are inversely related to miR-146a levels both *in vitro *and *in vivo*. Similar results were obtained from cultured human chondrocytes (data not shown). Mutation of the miR-146a binding site in the 3' UTR of Smad4 mRNA unequivocally identified Smad4 as a direct target of miR-146a for post-transcriptional regulation. Furthermore, miR-146a is critical for IL-1β downregulation of Smad4 in chondrocytes.

Our data suggest that miR-146a regulates chondrocytes and OA pathogenesis by inhibiting Smad4, a pivotal mediator of the TGF-β signaling pathway. Interestingly, the extent of miR-146a inhibition of Smad4 protein levels is more than the extent of miR-146a inhibition of Smad4 mRNA levels. This indicates that miR-146a targets Smad4 through both mRNA degradation and translational repression. Smad4 plays important roles in regulating chondrocyte differentiation by inhibiting hypertrophy and cell apoptosis. In the cartilage-specific Smad4 knockout mice, chondrocyte proliferation is reduced, hypertrophic differentiation is accelerated, and apoptosis is increased [35]. Furthermore, IL-1β inhibits Smad4 in a chondrocytic cell line (SW1353), indicating that the antagonistic effect of IL-1β on TGF-β may be mediated by blocking the expression of Smad4 [36]. TGF-β may counteract some IL-1β-induced effects on cartilage deterioration by preserving chondrocyte phenotypes, suppressing the expression of MMPs, such as MMP-1 and MMP-3, and promoting the synthesis of extracellular matrix of cartilage [37-39]. Loss of TGF-β and its downstream signaling molecules often corresponds with skeletal abnormalities and destruction of articular cartilage. For example, overexpression of a functionless TGF-β type II receptor accelerates terminal chondrocyte differentiation [40]. Moreover, Smad3 mutant mice display a phenotype resembling human OA, which is accompanied by the extensive progression of chondrocyte hypertrophy and osteophyte formation [41].

We demonstrate that miR-146a inhibits chondrocyte response to TGF-β by suppressing transcriptional activity of a promoter harboring TGF-β responsive elements and by suppressing TGF-β induction of ERK activity. The activation of ERK mitogen-activated protein kinases represents a downstream molecular event in response to TGF-β in chondro-progenitor cells, which is required for TGF-β-induced aggrecan expression [42]. ERK not only directly promotes phosphorylation of R-Smads, but also affects co-activators or co-repressors that mediate Smad DNA binding [43]. It has been shown previously that TGF-β stimulation of ERK activity is Smad4 dependent [44]. Knockdown of Smad4 by miR-146a may therefore inhibit ERK phosphorylation. Similar to miR-146a, other miRNAs have been implicated in regulating TGF-β pathways by targeting Smads in chondrocytes. For example, miR-199a* was reported to inhibit early chondrogenic differentiation by targeting Smad1 directly [45].

We demonstrate that miR-146a results in an increase of the apoptosis rate in articular chondrocytes. Reduced cellularity in articular cartilage contributes to the onset and development of OA. A higher proportion of apoptotic cells was observed in the cartilage from OA patients compared with that from normal people [46]. Expressions of apoptotic molecular markers, such as caspase-3 and caspase-8, were elevated in human osteoarthritic cartilage [47]. These are consistent with our hypothesis that miR-164a contributes to OA pathogenesis by inducing chondrocyte apoptosis.

Lastly, our data indicate that at least some of the effects of miR-146a on OA pathogenesis may be exerted by VEGF. We demonstrate that VEGF expression is upregulated by induction of OA pathogenesis with joint instability, treatment of IL-1β, overexpression of miR-146a, or knockdown of Smad4. Furthermore, induction of VEGF by IL-1β at least partially depends on upregulation of miR-146a; and its induction by miR-146a depends on Smad4 downregulation. Smad4 has been shown previously to inhibit VEGF expression and suppress tumorigenicity through inhibition of angiogenic activity in human pancreatic carcinoma cells [48]. Interestingly, while the miR-146a inhibitor significantly affects the IL-1β regulation of Smad4 and VEGF, inhibition of miR-146a could not completely eliminate IL-1β-caused stimulation of VEGF and suppression of Smad4. This suggests that, in addition to miR-146a, other factors are involved in mediating IL-1β regulation of VEGF and Smad4.

The induction of VEGF expression by miR-146a may affect angiogenesis and inflammation during OA pathogenesis. VEGF is increased in the osteoarthritic synovium [49] and OA cartilage [50]. Upregulation of VEGF contributes to inflammation and pathological angiogenesis in OA [51,52]. On the other hand, the upregulation of VEGF may also lead to chondrocyte hypertrophy, matrix degradation, and cell death - a series of critical events during endochondral ossification that is recapitulated during OA pathogenesis [53,54]. VEGF, upregulated by hypertrophic chondrocytes, may in turn induce the invasion of blood vessels to cartilage, secretion of MMPs, extracellular matrix remodeling, and, ultimately, cell death [55].

## Conclusions

We demonstrate that miR-146a may be involved in a novel signaling cascade critical for a series of IL-1β-induced pathologic features of OA including reduced cellular response to TGF-β, elevated VEGF expression, and increased chondrocyte apoptosis. Our results demonstrate for the first time that Smad4 is a direct target of miR-146a, and a critical mediator of miR-146a regulation of VEGF expression. Our results provide deeper insights into the roles of miRNA in OA pathogenesis and raise the possibility that miR-146a may be a therapeutic target for the treatment of OA.

## Abbreviations

BSA: bovine serum albumin; DMEM: Dulbecco's modified Eagle's medium; ERK: extracellular signal-regulated kinase; IL: interleukin; miRNA: microRNA; MMP: matrix metalloproteinase; OA: osteoarthritis; PBS: phosphate-buffered saline; PCR: polymerase chain reaction; RNAi: RNA interference; RT: reverse transcription; siRNA: small interfering RNA; TGF: transforming growth factor; TNF: tumor necrosis factor; TUNEL: terminal deoxynucleotidyl transferase dUTP nick end labeling; UTR: untranslated region; VEGF: vascular endothelial growth factor.

## Competing interests

The authors declare that they have no competing interests.

## Authors' contributions

JL participated in the design of the study, carried out the experiments and statistical analysis, and drafted the manuscript. JGH and LMD assisted in performing immunohistochemistry. DGY assisted with *in vivo *experiments. QC was involved in data interpretation and manuscript preparation. KRD and XLZ conceived of the study, participated in its design and coordination, and helped to draft the manuscript. All authors read and approved the final manuscript for publication.

## Supplementary Material

Additional file 1 **Figure S1 showing a heatmap of miRNA expression profiles of chondrocytes stimulated with IL-1β**. Statistically significant miRNAs (*P *< 0.01, selected by analysis of variance test) are presented. Green and red denotes downregulated and upregulated expression, respectively. Click here for file
