# Supplementary material for: miR-146a, an IL-1β responsive miRNA, induces vascular endothelial growth factor and chondrocyte apoptosis by targeting Smad4
Source: Arthritis Res Ther. 2012 Apr 16;14(2):R75. doi: 10.1186/ar3798 (PMC3446449; doi:10.1186/ar3798)
Supplement: Additional file 1 — Figure S1 showing a heatmap of miRNA expression profiles of chondrocytes stimulated with IL-1β. Statistically significant miRNAs (P < 0.01, selected by analysis of variance test) are presented. Green and red denotes downregulated and upregulated expression, respectively. [file ar3798-S1.PDF]

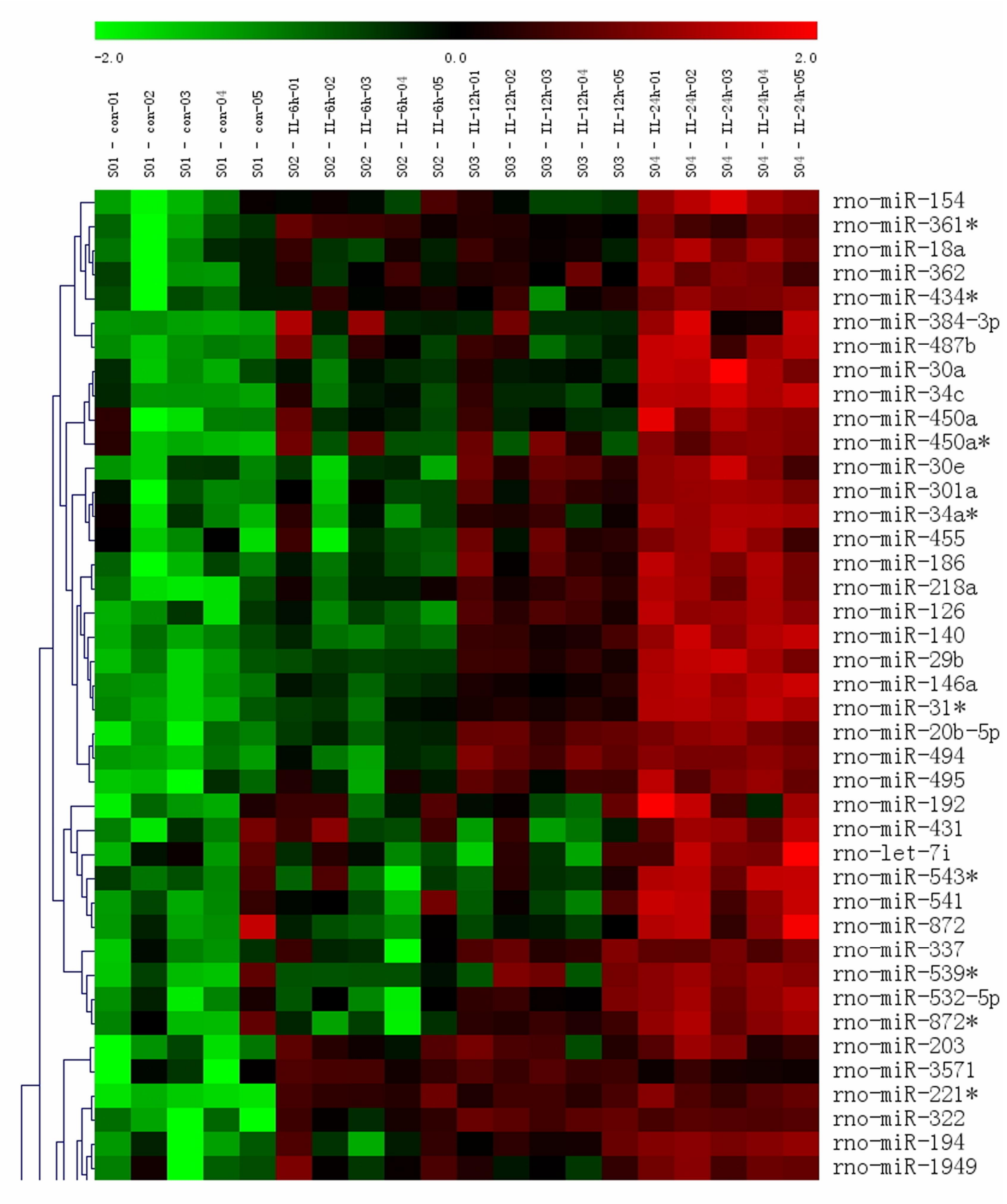

Figure S1. Heatmap of microRNA expression profiles of chondrocytes stimulated with IL-1β.

Statistically significant miRNAs (p-value < 0.01, selected by ANOVA test) are presented. Green and red color denotes down-regulated and up-regulated expression, respectively.
